# Triclosan promotes epicutaneous sensitization to peanut in mice

**DOI:** 10.1186/s13601-016-0102-2

**Published:** 2016-04-05

**Authors:** Steven Tobar, Leticia Tordesillas, M. Cecilia Berin

**Affiliations:** Pediatric Allergy and Immunology, Mindich Child Health and Development Institute, Icahn School of Medicine at Mount Sinai, One Gustave L. Levy Place, Box 1198, New York, NY 10029 USA

**Keywords:** Food allergy, Sensitization, Anaphylaxis, Peanut, Triclosan, Epicutaneous

## Abstract

**Background:**

Peanut allergy is increasing in prevalence due to unknown factors. A growing body of clinical evidence suggests sensitization to peanut occurs through the skin, supported by findings in mouse models. There is a need to identify environmental factors that promote epicutaneous sensitization to peanut. Triclosan is an antimicrobial found in household products that has been associated with food sensitization in humans. We tested the impact of triclosan on epicutaneous sensitization to peanut, as well as the milk allergen α-lactalbumin (ALA).

**Results:**

We observed that topical triclosan promoted epicutaneous sensitization to both peanut and ALA, and promoted anaphylaxis to peanut.

**Conclusions:**

Our results demonstrate that the mouse model of epicutaneous sensitization to foods is effective for demonstrating the clinically significant impact of environmental factors such as triclosan on food allergy.

## Rationale

IgE-mediated food allergy is becoming more prevalent in the western world. From 1997 to 2007, food allergy prevalence increased by 18 % among children under the age of 18 years old in the United States [1]. Estimates for the rise in peanut and tree nut allergy have been more dramatic, with a greater than threefold increase [2]. There is growing clinical evidence that sensitization to peanut occurs through the skin [3]. We recently showed in mice that epicutaneous peanut exposure could induce sensitization in the absence of adjuvant or skin damage [4]. We showed that peanut had innate adjuvant activity through activation of IL-33-dependent pathways. This epicutaneous sensitization model helps to explain why peanut is comprised of such potent allergens, but does not incorporate risk factors that increase individual susceptibility to peanut allergy.

Environmental factors that could contribute to the increasing prevalence of food allergy include common environmental chemicals. Triclosan, an antimicrobial agent found in consumer products such as hand sanitizer, soap, and shampoo, has been associated with food sensitization in humans [5]. Triclosan enhances sensitization in a model of experimental asthma [6]. Triclosan has also been shown to induce TSLP expression in the skin, and drive a Th2-skewed immune response [7]. We hypothesized that exposure to environmental factors, such as triclosan, could promote individual susceptibility to peanut allergy in the context of epicutaneous peanut exposure, and set out to test this in the mouse model of epicutaneous sensitization.

## Methods

### Reagents

Defatted crude peanut extract (CPE) was prepared from deshelled roasted peanuts that were ground to a paste, defatted with acetone, dried overnight, and extracted with PBS with protease inhibitor (Roche Life Science, Indianapolis, IN). α-Lactalbumin (ALA) and triclosan were purchased from Sigma-Aldrich (St. Louis, MO).

### Mouse model of sensitization

C3H/HeJ mice were acquired from the National Cancer Institute (Frederick, MD). Female mice (4–5 weeks old) were anesthetized, followed by exposure to 50 μL of (5, 50, or 500 μg) CPE or ALA (100 μg) with vehicle (acetone) or triclosan (1 %) on the ear pinnae. The top dose of CPE and the dose of ALA were previously found to induce sensitization without (CPE) and with (ALA) adjuvant [4]. Mice were exposed weekly for 6 exposures, and mice were challenged 1 week after the last exposure. All procedures were approved by the Animal Care and Use Committee of the Icahn School of Medicine at Mount Sinai (Protocol LA11-00273).

### Allergen challenge and anaphylaxis measurement

Mice were challenged by intraperitoneal injection with 100 μg of CPE, and were observed following challenge for 30 min. Mice were orally challenged with 50 mg of ALA, followed by intraperitoneal challenge with 50 μg of ALA in those that did not react. In our experience, mice sensitized to ALA will respond to oral challenge while mice sensitized to peanut require systemic (ip) challenge to induce anaphylaxis. Body temperature was recorded before and 30 min after challenge by rectal thermometer (WPI Instruments, Sarasota, FL).

### Antibody measurement

Allergen-specific IgE, IgG1, and IgG2a were measured in serum obtained prior to challenge. IgE was measured by coating ELISA plates with monoclonal anti-IgE (BD Biosciences, Franklin Lakes, NJ) and detecting with DIG-labeled CPE or ALA. IgG1 and IgG2a were measured by directly coating allergens on the plate and detecting with biotinylated anti-IgG1 or IgG2a (BD Biosciences), followed by Avidin-HRP and TMB reagent (eBiosciences, San Diego, CA). A SpectraMax plate reader with background subtraction was used to measure absorbance (450 nm).

### Statistics

Differences between groups were measured by ANOVA, followed by post hoc testing with non-parametric T test (GraphPad Prism, La Jolla, CA).

## Results

### Impact of triclosan on dose-dependent epicutaneous sensitization of mice to peanut

**Fig. 1:**
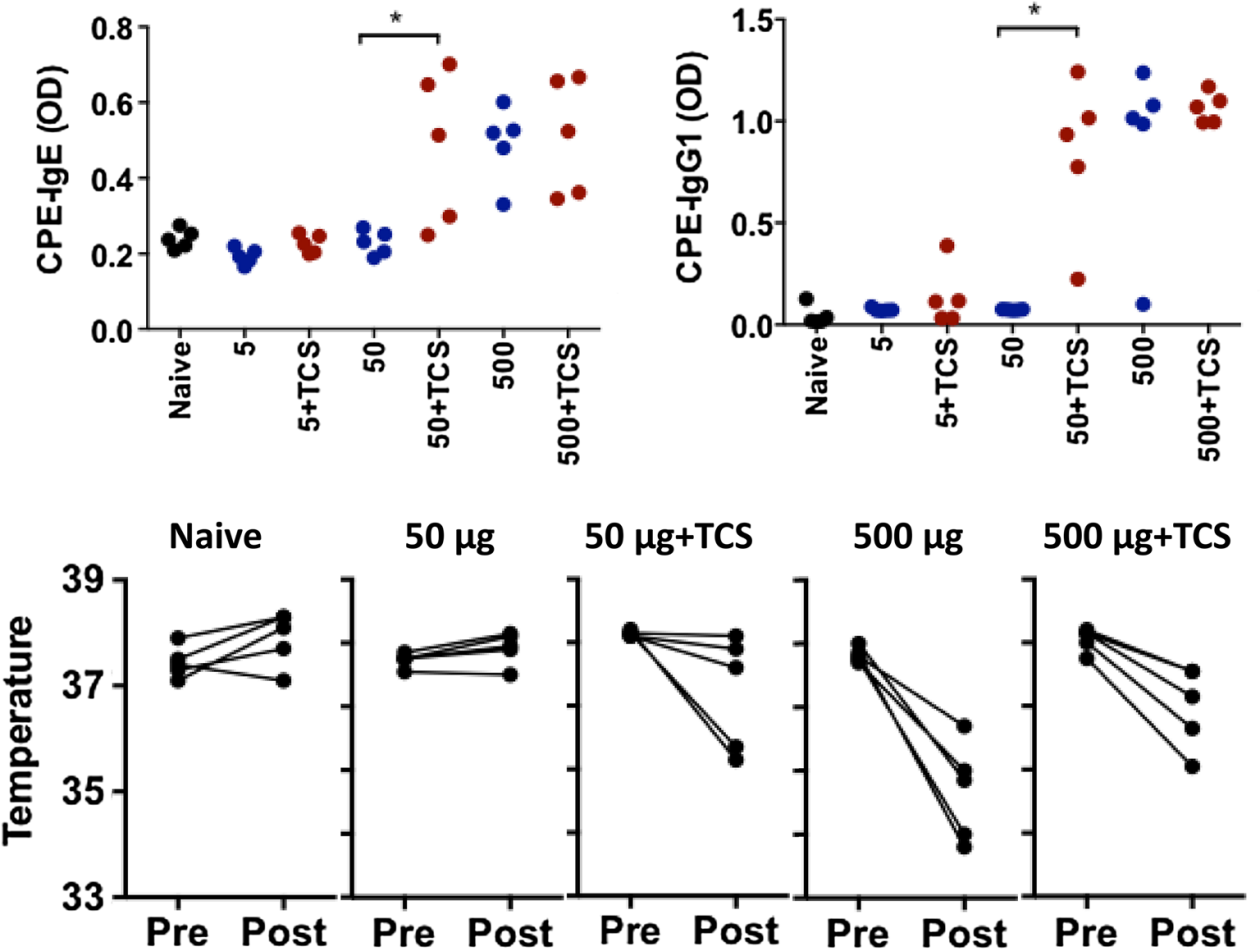
Impact of triclosan on peanut allergy. Mice were left naïve or exposed to 5, 50, or 500 μg of CPE in vehicle (−TCS) or 1 % triclosan (+TCS) once a week for 6 weeks. *Panels* show CPE-specific IgE, and IgG1 (*top*), and anaphylaxis measured by drop in body temperature measured prior to challenge (*pre*) and 30 min after challenge (*post*). *p < 0.05 compared to the −TCS control

We previously showed that mice exposed to 1 mg of CPE dissolved in PBS via the epicutaneous route develop IgE sensitization and anaphylaxis to peanut [4]. Mice were epicutaneously exposed to 5, 50, or 500 μg of CPE with and without triclosan on the ear pinnae once a week for 6 weeks. Triclosan was dissolved in acetone, and therefore acetone was used as control vehicle. Mice exposed to 500 μg of CPE produced peanut-specific IgE and IgG1 antibodies and 100 % of mice experienced anaphylaxis when challenged with peanut (Fig. 1). Mice exposed to 50 or 5 μg of CPE did not sensitize to peanut. The presence of triclosan at the 500 μg dose of CPE did not influence CPE-specific IgE, IgG1, or IgG2a levels or CPE-induced anaphylaxis. However, at the 50 μg dose of CPE, triclosan significantly increased CPE-specific IgE and IgG1 compared to the naïve or peanut alone groups (Fig. 1). There was also a trend toward decreased CPE-specific IgG2a in the presence of triclosan (data not shown). Consistent with this increase in peanut-specific IgE, a subset of mice treated with triclosan and 50 μg CPE developed anaphylaxis after peanut challenge (Fig. 1). Triclosan did not induce sensitization when given with the lowest dose (5 μg) of CPE.

### Impact of triclosan on epicutaneous sensitization to the milk allergen α-lactalbumin

**Fig. 2:**
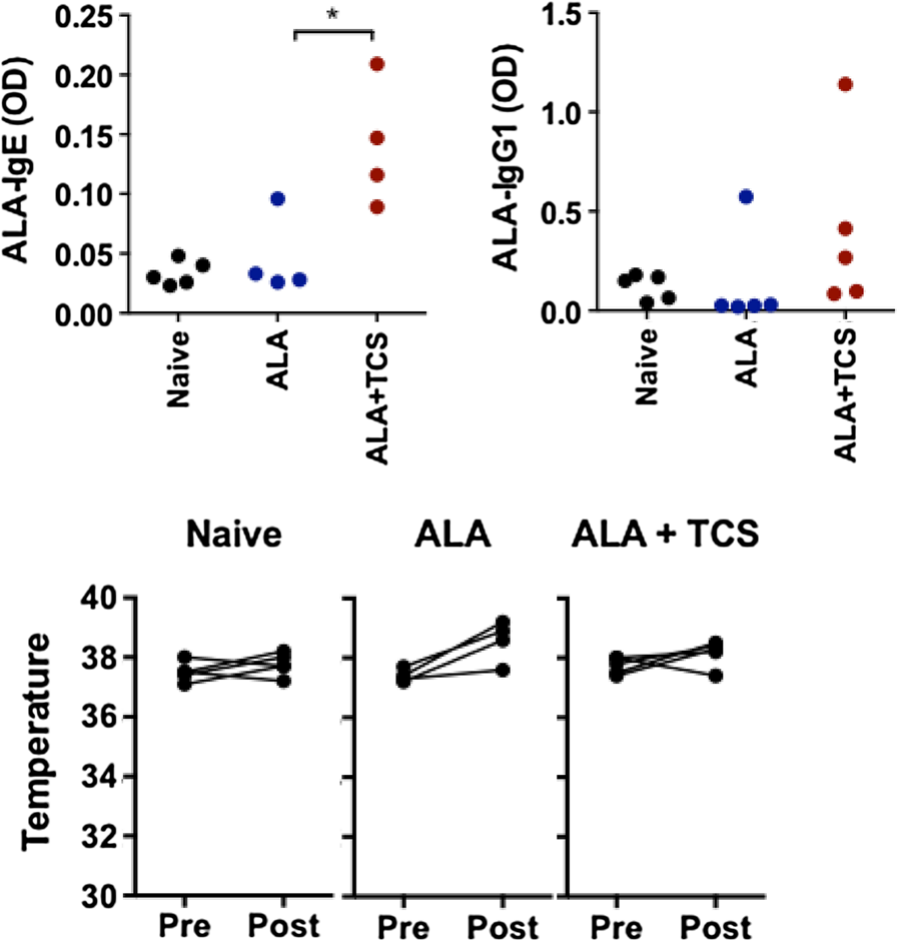
Impact of triclosan on sensitization to α-lactalbumin (ALA). Mice were left naïve (n = 5), or exposed to 100 μg of ALA in vehicle (−TCS, n = 4) or 1 % triclosan (+TCS, n = 5) once a week for 6 weeks. Panels show ALA-specific IgE and IgG1 (*top*), and anaphylaxis assessed by drop in body temperature measured prior to challenge (*pre*) and 30 min after challenge (*post*). *p < 0.05 compared to the −TCS control

Mice exposed to α-lactalbumin (ALA) develop sensitization to ALA only in the presence of an adjuvant such as cholera toxin or SEB [4, 8], indicating a lack of inherent adjuvant activity in this milk allergen. To determine if triclosan can also promote sensitization to allergens without inherent adjuvant activity, mice were exposed to ALA in the presence of triclosan or vehicle control once a week for 6 weeks. Similar to previous results, mice exposed to ALA alone did not develop sensitization, nor did they have an anaphylactic response. Mice exposed to ALA in the presence of triclosan developed ALA-specific IgE and IgG1 (Fig. 2). ALA-specific IgG2a was undetectable in all conditions (not shown). In this case, the enhancement of ALA-specific sensitization by triclosan was not sufficient to induce anaphylaxis (Fig. 2).

## Discussion

With the prevalence of food allergies increasing to as much as 10 % in the developed world [9], understanding the underlying cause(s) driving this increase is imperative. Triclosan is extensively used in household consumer products, and is found in more than 75 % of the US population [10] and is one of the top ten environmental contaminants of American rivers [11]. Here we show that topical application of triclosan had a modest but significant effect on sensitization associated with experimental food allergy. Triclosan reduced the exposure level required to induce sensitization to allergens with inherent adjuvant activity (peanut), and functioned as an adjuvant when used with weaker allergens (ALA).

Epithelial-derived Th2-inducing cytokines such as TSLP and IL-33 play an important role in driving sensitization to foods [4, 12]. We previously showed that peanut could induce production of IL-33 in the skin [4], while triclosan was previously shown to induce induce TSLP [7]. Triclosan also suppressed IL-33 expression in skin, indicating that it may counteract the effect of peanut. However, in combination we see that triclosan significantly promotes the development of IgE sensitization to peanut.

This brief report extends our previous detailed description of the epicutaneous sensitization model [4] by demonstrating the utility of the model in studying the impact of environmental factors on susceptibility to sensitization. Our data also importantly provide evidence of causality in the finding in human cohorts that elevated urinary triclosan is associated with food sensitization [5]. The increasing prevalence of peanut allergy is likely due to multiple factors that individually have modest but biologically significant effects. Environmental factors, either single factors or in combination, can be tested for their potential impact on susceptibility to food allergy using this clinically relevant model of sensitization to peanut.

## Abbreviations

CPE: crude peanut extract
ALA: α-lactalbumin
